# Sarcopenia Is Negatively Related to High Gravitational Impacts Achieved From Day-to-day Physical Activity

**DOI:** 10.1093/gerona/glx223

**Published:** 2017-11-22

**Authors:** April Hartley, Celia L Gregson, Kimberly Hannam, Kevin C Deere, Emma M Clark, Jon H Tobias

**Affiliations:** 1 Musculoskeletal Research Unit, Translational Health Sciences, Bristol Medical School, University of Bristol, UK; 2 Integrative Epidemiology Unit, Population Health Sciences, Bristol Medical School, University of Bristol, UK

**Keywords:** Muscle function, Physical capability, Accelerometer, G-force, COSHIBA

## Abstract

**Background:**

Sarcopenia has been associated with reduced physical activity (PA). We aimed to determine if sarcopenia, and specific components of muscle size, function, and physical performance, are associated with high impacts achieved during habitual PA, as these are related to bone strength in community-dwelling older women.

**Methods:**

Participants were older women from the Cohort of Skeletal Health in Bristol and Avon. We defined sarcopenia using the EWGSOP criteria. Lower limb peak muscle power and force were assessed using Jumping Mechanography (JM). High vertical impacts were assessed by tri-axial accelerometry (at least 1.5g above gravity). Cross-sectional associations were analyzed by linear regression, adjusting for age, height and weight (or fat mass for models including appendicular lean mass index), comorbidities, smoking, alcohol, and Index of Multiple Deprivation.

**Results:**

Our analyses included 380 participants, with mean age 76.7 (*SD* 3.0) years; 242 (64%) also completed JM. In age-adjusted analysis, a negative relationship was observed between severity of sarcopenia and high, but not medium or low, impacts (*p* = .03 for trend). Regarding components of sarcopenia underlying this relationship, multivariable analyses revealed that gait speed (β 1.47 [95% CI 1.14, 1.89], [β-1] reflects the proportionate increase in high impacts per *SD* increase in exposure) and peak force (1.40 [1.07, 1.84]) were independently associated with high impacts.

**Conclusions:**

Older women with sarcopenia experienced fewer bone-strengthening high impacts than those with presarcopenia or without sarcopenia. To increase bone strengthening activity in older women, interventions need to improve both lower limb muscle force and walking speed.

Sarcopenia, a common disorder of older age, is associated with increased mortality (1) and constitutes a significant economic burden (2). The European Working Group on Sarcopenia in Older People (EWGSOP) defines sarcopenia as low Appendicular Lean Mass Index (ALMI) plus low muscle strength and/or low physical performance (3). Additional categories comprise presarcopenia (low ALMI only), and severe sarcopenia (low ALMI, muscle strength, and performance). Prevalence estimates for sarcopenia vary depending upon the definition, with recent estimates suggesting up to 29% of community-dwelling older adults are sarcopenic (4). Frailty, one of the main clinical manifestations of sarcopenia, partly reflects the functional consequences of impaired muscle strength on physical performance. For example, the widely used short physical performance battery (SPPB, based on gait speed, chair-rise time, and balance (5)) can be used to identify individuals with sarcopenia and frailty (3,6).

Sarcopenia has been associated with reduced PA; sarcopenic adults (defined using EWGSOP criteria) participated in less moderate-vigorous PA (MVPA) than nonsarcopenic adults in a recent large population-based analysis (7). Longitudinal studies suggest being more active may prevent age-related loss of lean mass (8,9). Conversely, recent longitudinal analyses from UK Biobank identified a positive relationship between baseline grip strength and PA at follow-up (10), suggesting that better muscle function also predicts ability to undertake PA. Further evidence exists supporting a cross-sectional association between physical activity (PA) and physical performance (11–14).

Interest is growing regarding the influence of vertical impacts achieved during habitual PA, particularly on Bone Mineral Density (BMD), since historic animal studies first highlighted the relationship between mechanical strain and bone formation (15). A meta-analysis of intervention studies for postmenopausal women found that interventions including high impact PA (eg, jogging), combined with walking and stair climbing, were most beneficial at preserving hip and lumbar spine BMD (16). Moreover, a hopping intervention successfully increased BMD of the femoral neck in older men (17). Furthermore, we recently determined that exposure to high impacts (at least 1.5g above gravity), ascertained by 7-day accelerometry, was associated with hip strength in community-dwelling older women (18).

Although sarcopenia has been associated with lower generalized PA (7), the extent to which PA of differing impacts is specifically reduced has not been evaluated. As high impact PA places a greater demand on muscle function, we hypothesized that sarcopenia would be strongly associated with reduced high impacts. Therefore, we aimed to determine the relationship between sarcopenia and habitual levels of PA, categorized according to levels of vertical impact, in a population of community-dwelling older women. Furthermore, we examined associations between different components of muscle size, function, and physical performance and vertical impacts, including lower limb muscle force, which we hypothesize to be particularly strongly related to high levels of vertical impacts.

## Methods

### Study Population

**Figure 1.**
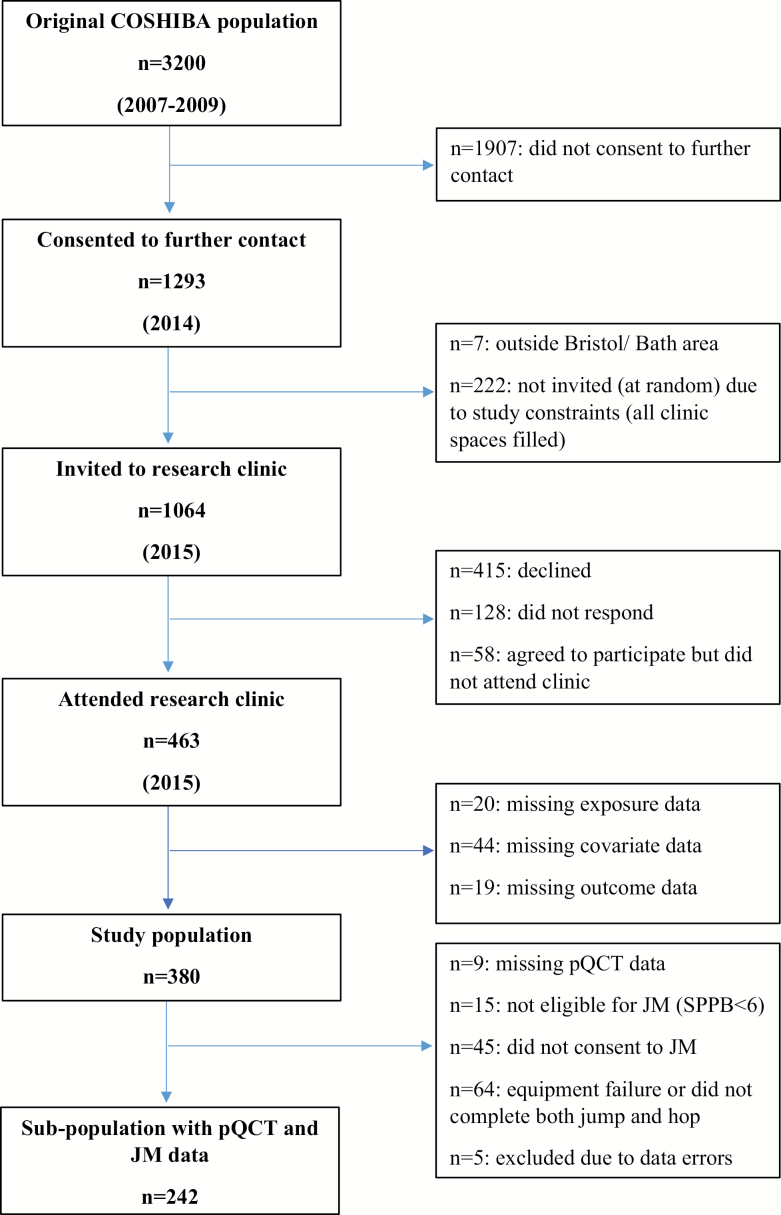
Flowchart of participant recruitment process and selection of study population. COSHIBA = Cohort of Skeletal Health in Bristol and Avon; pQCT = Peripheral Quantitative Computed Tomography; JM = Jumping Mechanography.

Participants were recruited from the Cohort of Skeletal Health in Bristol and Avon (COSHIBA), comprising women recruited during 2007–2009 through general practitioner registries within Southwest England, born between January 1, 1927 and December 31, 1942 (19). Four hundred and sixty-three women were recruited 5–7 years from baseline (14% of baseline), who attended our research clinic for musculoskeletal and physical function assessments during 2015 (Figure 1). A detailed explanation of all assessments was provided and full written consent obtained. Participants completed a study questionnaire after functional assessment, collecting demographic, health, and lifestyle data. The study was approved by the South West: Frenchay Research Ethics Committee (14/SW/0138).

### Exposures

#### Jumping mechanography (JM)

Muscle power and force were assessed using a Leonardo Mechanography Ground Reaction Force platform. The platform consists of two plates with corner sensors which detect voltage proportional to applied force (20). Sensor recordings are used to derive test-specific performance calculations using Leonardo software (version 4.2, Novotec Medical, Germany). All participants with a SPPB (see next methods section) score ≥6 were eligible (ie, physically capable and safe to jump). Peak power was assessed by two-legged jump and peak force by one-legged hopping. Additional information is provided in the Supplementary Material.

#### SPPB

SPPB comprises tests of balance (side-by-side, semitandem, and tandem balances performed for up to a maximum of 10 seconds), chair rise time (five timed chair rises as fast as possible [without using arms]), and gait speed (4-m timed walk at usual speed, best of two scored), each test being scored out of four points giving a maximal total score of 12 (5).

#### Dual x-ray absorptiometry (DXA) and anthropometric measures

All consenting participants underwent DXA scanning of the total body to measure lean mass (LM) and fat mass (FM); the methodology has previously been described (18) (see Supplementary Material). Before DXA scanning, height was measured using a Harpenden stadiometer (Holtain Ltd., Crymych, UK), to the nearest millimetre. Weight was measured using Tanita scales (Tanita UK Ltd., Uxbridge, UK), to the nearest 0.5 kg. ALMI was calculated as (total LM arms + total LM legs)/height^2^.

#### Peripheral quantitative computed tomography (pQCT) measured muscle size

The methodology for pQCT acquisition is described elsewhere (18) (see Supplementary Material). Muscle cross-sectional area (mCSA), measured at 50% of tibial length, was derived as the total limb cross-sectional area with density ≥50 mg/cm^3^ minus total bone content, applied filters derived smoothed total mCSA.

#### Upper limb grip strength

Grip strength was assessed using a JAMAR digital handgrip dynamometer (Patterson Medical, IL), with the handle set at the second point. Three tests were performed on each hand (with 30 seconds rest between each test) with the participant standing upright (unless unable to stand) with their arm fully extended next to their body and a stiff wrist. The participant was instructed to squeeze as hard as they can for as long as they can, using standard instructions. Measurements alternated between each hand with a total of three attempts for each hand and the maximal value was taken. Measurements were recorded to the nearest 0.1 kg.

#### PA outcomes

The protocol for PA monitoring, and derivation of impact variables, has been described elsewhere (21). Briefly, participants were given a Gulf Coast Data Concepts x16-1c tri-axial accelerometer (Waveland, MS) to wear in a custom-made size-specific elastic belt over their right hip during all waking hours (excluding swimming and washing) for 7 days. The accelerometers were configured prior to each use with a sampling frequency of 50 Hz, a deadband setting of 0.1 g (threshold to be exceeded for a recording to be made) and a timeout setting of 10 seconds (time of inactivity before the monitor turned itself off to conserve battery life). A timesheet was provided for the participant to record the time they wore the monitor each day, as well as any comments (eg, monitor removed at *x* time for swimming). Data was uploaded and imported into Stata (StataCorp, TX) and processed using custom-designed code which cleaned data to remove artifacts (eg, monitor falling out of belt) and nonwear time. A valid day was defined as at least 10 hours of wear time. A peak was identified as any vertical acceleration greater than the previous or subsequent recorded acceleration. Peaks were classified into three impact bands: low (0.5≤g<1.0), medium (1.0≤g<1.5), and high (≥1.5 g), over and above Earth’s gravity. The number of counts in each impact band were normalized to 7 days of 14 hours.

### Statistical Analysis

Presarcopenia was defined as low muscle mass (ALMI ≤ 5.45 kg/m^2^), sarcopenia as low muscle mass and low grip strength (<20 kg) or gait speed (<0.8 m/s), and severe sarcopenia as low muscle mass, grip strength, and gait speed (3). Due to small numbers in the severe sarcopenia category, sarcopenia and severe sarcopenia were combined for analyses.

Descriptive statistics are presented as counts and percentages for categorical variables, and mean (standard deviation [*SD*]) for continuous variables, except for accelerometry counts which were positively skewed and are presented as median (interquartile range [IQR]), and log-transformed.

Relationships between sarcopenia/individual components of muscle size and function, and accelerometry counts (number of vertical accelerations) in each PA impact band, were examined using linear regression. We adjusted for the following a priori confounders: age at clinic session (model 1); model 1 plus height and weight (except for sarcopenia based on ALMI when total body FM was included instead of height and weight) (model 2); model two plus Index of Multiple Deprivation (IMD), smoking, alcohol consumption (0, >0–<10 or ≥10 units), and comorbidities (0, 1 or ≥2) (model 3). Methods regarding acquisition of covariates are described in full in the Supplementary Material. To identify independent determinants of accelerometry counts, muscle function variables were included in the same multivariable model, and model fit subsequently evaluated (*r*^2^ values and Akaike’s Information Criterion [AIC]). All continuous exposure variables were standardized. As outcome variables were log-transformed, coefficients represent the percentage change in outcome per *SD* change in exposure, and percentage difference in geometric mean for categorical variables. Data analysis was performed using Stata version 13 (StataCorp, TX). Sensitivity analyses are described in the Supplementary Material.

## Results

### Participant Characteristics

One thousand sixty-four members of the COSHIBA population were invited to reattend of whom 380 (36%) provided complete data for SPPB, sarcopenia exposures, covariates, and PA outcomes and formed the study population (Figure 1). Compared to the baseline COSHIBA population, those included in the present analysis were younger, taller, had a lower BMI and were more highly educated (Supplementary Table 1).

The mean age of those included in the present study was 76.7 (3.0) and the mean BMI 27.0 (4.6) kg/m^2^ (Table 1). 6.3% were classified as presarcopenic, 3.7% as sarcopenic, and 1.8% as severely sarcopenic. The population experienced a median of 8,838 (IQR 4,333, 16,934) low impacts, 345.3 (109.2, 742.5) medium impacts, and 41.8 (17.2, 105.9) high impacts over 1 week. The subgroup who underwent JM had a mean age of 76.4 (2.6), mean BMI 26.0 (3.8) kg/m^2^ and 7.4%, 4.1%, and 0.8% were categorized as presarcopenic, sarcopenic, and severely sarcopenic, respectively.

**Table 1.** Descriptive Characteristics of the Study Population and JM Subpopulation

		Overall Study Population (*n* = 380)	Subgroup Having JM (*n* = 242)
		Mean (*SD*)	Mean (*SD*)
Age		76.7 (3.0)	76.4 (2.6)
Alcohol (units in past week)^a^		5.4 (7.4)	6.3 (7.5)
IMD Rank		19,734 (8,970)	20,340 (8,872)
BMI (kg/m^2^)		27.0 (4.6)	26.0 (3.8)
TBFM (kg)		27.9 (8.4)	26.4 (7.3)
SPPB score^b^		10.0 (2.0)	10.7 (1.3)
Gait speed (m/s)		1.0 (0.2)	1.1 (0.2)
Chair rise time (s)		13.6 (5.0)	12.9 (4.2)
ALMI (kg/m^2^)		6.5 (1.0)	6.3 (0.8)
mCSA (mm^2^)			4,058.4 (855.8)
Peak power (kW)			1.4 (0.3)
Peak force (kN)			1.3 (0.2)
Grip strength (kg)		21.1 (5.1)	21.8 (4.9)

		Median (IQR)	Median (IQR)
Physical activity	Low impact counts^c^	8,837.9 (4,332.9, 16,934.4)	11,457.8 (5,779.1, 18,827.9)
	Medium impact counts^c^	345.3 (109.2, 742.5)	452.6 (183.7, 950.9)
	High impact counts^c^	41.8 (17.2, 105.9)	51.8 (23.0, 124.2)

		*N* (%)	*N* (%)
Ethnicity	White British^a^	368 (96.8)	237 (97.9)
Comorbidities	0	148 (39.0)	111 (45.9)
	1	173 (45.5)	107 (44.2)
	≥2	59 (15.5)	24 (9.9)
Smoker^a^	Never	231 (60.8)	152 (62.8)
	Current	4 (1.1)	3 (1.2)
	Past	145 (38.2)	87 (36.0)
Calcium/Vitamin D supplements^a^		142 (37.4)	76 (31.4)
Tandem balance	Held < 10 s	84 (22.1)	29 (12.0)
Sarcopenia^d^	None	335 (88.2)	212 (87.6)
	Presarcopenia	24 (6.3)	18 (7.4)
	Sarcopenia	14 (3.7)	10 (4.1)
	Severe	7 (1.8)	2 (0.8)

*Note*: ALMI = Appendicular lean mass index; BMI = Body mass index; IMD = Index of multiple deprivation; IQR = Interquartile range; mCSA = Muscle cross-sectional area; SD = Standard deviation; TBFM = Total body fat mass.

a Self-reported in study questionnaire.

b SPPB = Short physical performance battery. Score out of 12 based on a maximum of four for each of component: balance, chair rise and gait speed

c Low impacts 0.5≤0.5 g<1.0 g, medium impacts 1.0≤g<1.5, high impacts ≥1.5 g

d Presarcopenia defined as ALMI ≤5.45 kg/m^2^. Sarcopenia defined as ALMI ≤5.45 kg/m^2^ plus either grip strength <20 kg or gait speed <0.8 m/s. Severe sarcopenia defined as ALMI ≤5.45 kg/m^2^, gait speed<0.8 m/s, and grip strength <20 kg.

Mean height and weight of overall population: 158.9 (6.1) cm, 68.2 (11.9) kg and of subpopulation: 159.7 (6.1) cm, 66.3 (10.2) kg.

### Clinical Sarcopenia Versus Accelerometry Impact Counts

**Figure 2.**
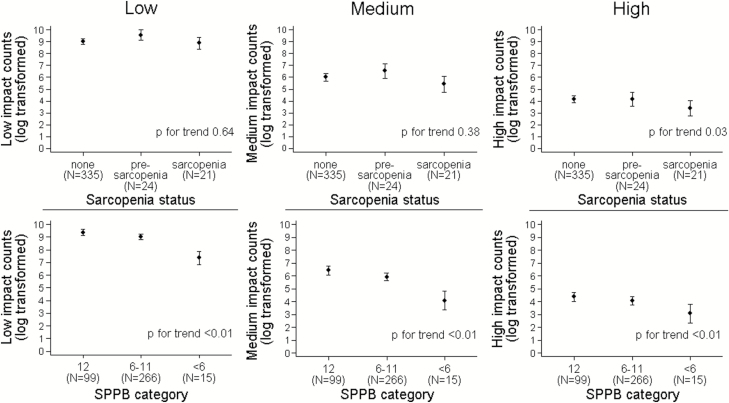
Plot of age-adjusted mean log-transformed impact counts by sarcopenia category and SPPB category. Points represent beta coefficients from linear regression adjusted for age. Bars represent 95% confidence intervals. Presarcopenia defined as ALMI ≤5.45 kg/m^2^. Sarcopenia defined as ALMI ≤5.45 kg/m^2^ plus either grip strength <20 kg and/or gait speed <0.8 m/s. ALMI = Appendicular Lean Mass Index; SPPB = Short Physical Performance Battery. Score out of 12 based on a maximum of four points for each of the three components: balance, chair rise, and gait speed. Low impacts 0.5<0.5g<1.0g, medium impacts 1.0<g<1.5, high impacts≥1.5g.

Sarcopenia status was unrelated to low or medium impacts; however, sarcopenia status was inversely associated with high impacts in our age-adjusted analyses, with lower levels seen in sarcopenia, whereas little difference was observed in presarcopenia (*p* for trend = .03; Figure 2). Decreased physical performance, as indicated by a lower SPPB score, was inversely associated with low, medium, and higher impacts, primarily reflecting decreased PA levels in those with significant physical frailty (SPPB score <6) (all *p* < .01).

In analyses examining differences in impact counts between those above and below the grip strength, gait speed and ALMI thresholds for defining sarcopenia, grip strength below 20 kg and gait speed slower than 0.8 m/s were associated with fewer low, medium, and high impacts over 1 week in all models (all *p* < .05), including our fully-adjusted model (model 3) (Supplementary Table 2). ALMI ≤5.45 kg/m^2^ was associated with approximately a 50% reduction in high impacts in models 2 and 3, but was not associated with low or medium impact counts in any model. Similar associations between low grip strength, gait speed and ALMI and impact counts were observed after additional adjustment for calcium/vitamin D supplementation (data not shown).

### Individual Components of Muscle Size/Function Versus Accelerometry Impact Counts

When gait speed was included as a continuous variable, similar associations were observed, with positive associations for all three impact categories and models (Table 2). Chair rise time was inversely related to low and medium impacts in all three models, but there was weaker evidence for an association with high impacts. Grip strength was positively related to low and medium impacts in models 2 and 3 and with high impacts in model 3. Peak force was positively related to low, medium, and high impacts in model 2 and 3. Peak power was positively related to low impacts (model 2 only), and to medium impacts (models 2 and 3), whereas no association was seen with high impacts. Tandem balance and mCSA were unrelated to impacts.

**Table 2.** Regression Analyses Showing the Relationships Between Measures of Muscle Function or Mass and Low, Medium, and High Impact Accelerometry Counts

		Model 1		Model 2		Model 3	
		β (95% CI)	*p*	β (95% CI)	*p*	β (95% CI)	*p*
Low	Gait speed	1.43 (1.25, 1.63)	<.01	1.33 (1.17, 1.52)	<.01	1.31 (1.14, 1.50)	<.01
	Chair stand time	0.80 (0.70, 0.91)	<.01	0.85 (0.75, 0.96)	<.01	0.87 (0.76, 0.99)	.04
	Tandem balance <10 s^a^	0.76 (0.54, 1.08)	.12	0.82 (0.59, 1.14)	.24	0.89 (0.63, 1.24)	.48
	Grip strength	1.09 (0.97, 1.23)	.14	1.14 (1.01, 1.28)	.04	1.15 (1.02, 1.30)	.03
	Peak Force	1.05 (0.90, 1.22)	.53	1.30 (1.11, 1.52)	<.01	1.25 (1.06, 1.47)	<.01
	Peak Power	0.97 (0.83, 1.13)	.71	1.18 (1.00, 1.39)	.04	1.15 (0.98, 1.36)	.09
	mCSA	0.92 (0.82, 1.03)	.15	1.02 (0.91, 1.15)	.74	1.02 (0.91, 1.15)	.73
Medium	Gait speed	1.93 (1.60, 2.33)	<.01	1.84 (1.52, 2.22)	<.01	1.83 (1.50, 2.22)	<.01
	Chair stand time	0.69 (0.57, 0.83)	<.01	0.72 (0.60, 0.87)	<.01	0.73 (0.60, 0.89)	<.01
	Tandem balance <10 s^a^	0.65 (0.40, 1.08)	.10	0.69 (0.42, 1.14)	.15	0.73 (0.44, 1.21)	.21
	Grip strength	1.15 (0.97, 1.37)	.11	1.21 (1.01, 1.45)	.03	1.25 (1.04, 1.51)	.02
	Peak Force	1.18 (0.95, 1.47)	.14	1.51 (1.19, 1.92)	<.01	1.44 (1.12, 1.84)	<.01
	Peak Power	1.14 (0.91, 1.42)	.26	1.46 (1.14, 1.86)	<.01	1.42 (1.11, 1.82)	<.01
	mCSA	1.01 (0.85, 1.20)	.92	1.13 (0.95, 1.35)	.17	1.13 (0.94, 1.35)	.19
High	Gait speed	1.53 (1.25, 1.87)	<.01	1.51 (1.22, 1.86)	<.01	1.56 (1.26, 1.94)	<.01
	Chair stand time	0.83 (0.68, 1.00)	.06	0.84 (0.69, 1.03)	.10	0.83 (0.67, 1.02)	.07
	Tandem balance <10 s^a^	0.83 (0.49, 1.39)	.47	0.85 (0.50, 1.43)	.54	0.82 (0.48, 1.40)	.46
	Grip strength	1.14 (0.95, 1.36)	.16	1.18 (0.97, 1.42)	.09	1.22 (1.01, 1.49)	.04
	Peak Force	1.26 (1.00, 1.57)	.05	1.46 (1.14, 1.88)	<.01	1.50 (1.15, 1.95)	<.01
	Peak Power	1.08 (0.86, 1.36)	.49	1.22 (0.94, 1.58)	.13	1.22 (0.93, 1.59)	.15
	mCSA	1.05 (0.88, 1.25)	.56	1.12 (0.93, 1.34)	.25	1.11 (0.92, 1.34)	.29

*Note*: Coefficients represent the change in low, medium or high impact counts for an *SD* increase in muscle function or size exposure (ie, a coefficient of 1.43 represents a 43% increase in low impact counts for a 1 *SD* increase in gait speed). Model 1: adjusted for age; Model 2: adjusted for age, height and weight; Model 3: adjusted for age, height, weight, smoking, alcohol, Index of Multiple Deprivation, and comorbidities. *N* = 242. CI = Confidence interval; mCSA = Muscle cross-sectional area.

a Coefficient represents the ratio of geometric means with tandem balance 10 s as the reference category.

Low impacts 0.5≤0.5 g<1.0 g, medium impacts 1.0≤g<1.5, high impacts≥1.5 g.

Multivariable linear regression analyses were performed with all predictor variables, based on model 3, to determine which measures of muscle size and function were independently associated with impacts. Gait speed was the only measure associated with low and medium impacts (1.26 [1.08, 1.48] and 1.68 [1.33, 2.12], respectively) (Table 3), whereas, gait speed and peak force were both independently associated with high impacts (1.47 [1.14, 1.89] and 1.40 [1.07, 1.84], respectively). Additional adjustment for calcium/vitamin D supplementation did not attenuate these relationships (data not shown). Together, a priori confounders (age, height, weight, IMD, comorbidities, smoking, and alcohol) explained 9% of the variance in high impacts, gait speed an additional 7%, and peak force an additional 4% (Supplementary Table 3). The high impact model comprising covariates, gait speed, and peak force had the best fit based on AIC, and explained the largest amount of total variance (*r*^2^ = 0.18).

**Table 3.** Multivariable Linear Regression Analyses Showing the Relationships Between Measures of Muscle Mass and Function and Low, Medium, and High Impact Accelerometry Counts

	Low		Medium		High	
	β (95% CI)	*p*	β (95% CI)	*p*	β (95% CI)	*p*
Gait speed	1.26 (1.08, 1.48)	<.01	1.68 (1.33, 2.12)	<.01	1.47 (1.14, 1.89)	<.01
Chair stand time	1.00 (0.87, 1.16)	.99	0.95 (0.77, 1.18)	.64	1.03 (0.82, 1.30)	.78
Tandem balance <10 s^a^	1.05 (0.75, 1.47)	.79	1.01 (0.62, 1.66)	.95	1.05 (0.61, 1.79)	.86
Grip strength	1.10 (0.96, 1.25)	.17	1.08 (0.89, 1.31)	.42	1.14 (0.92, 1.40)	.22
Peak force	1.17 (0.99, 1.39)	.07	1.23 (0.95, 1.57)	.11	1.40 (1.07, 1.84)	.02
Peak power	1.01 (0.84, 1.22)	.90	1.10 (0.84, 1.44)	.50	0.93 (0.69, 1.26)	.64
mCSA	0.95 (0.84, 1.08)	.42	0.96 (0.80, 1.15)	.64	1.01 (0.82, 1.23)	.94

*Note*: Exponentiated coefficients are presented representing the change in low, medium or high impact counts for an *SD* increase in muscle function or size exposure (eg, a coefficient of 1.24 represents a 24% increase in low impact counts for a 1 *SD* increase in gait speed). Adjusted for age, height, weight, smoking, alcohol, IMD, and comorbidities. *N* = 242.

CI = Confidence interval; mCSA = Muscle cross-sectional area.

a Coefficient represents the ratio of geometric means with tandem balance 10 s as the reference category.

Low impacts 0.5≤0.5 g<1.0 g, medium impacts 1.0≤g<1.5, high impacts ≥1.5 g

## Discussion

We investigated relationships between sarcopenia and habitual levels of PA, categorized according to level of vertical impact, in a community-based population of older women. We found that sarcopenia, defined using EWGSOP criteria, was associated with fewer high, but not low or medium, vertical impacts. In contrast, physical capability, as reflected by SPPB score, was related to low, medium, and high impacts. To clarify these relationships, we examined associations between individual components of sarcopenia and vertical impacts, with a focus on lower limb muscle function. Gait speed was independently associated with low, medium, and high impacts; peak lower limb muscle force was independently associated with high impacts only. Our results are broadly consistent with previous reports that sarcopenic individuals participated in less MVPA (7). However, we extend this by establishing relationships between sarcopenia and high impacts specifically.

There is significant overlap in the definitions of sarcopenia and physical frailty—gait speed and grip strength assessment can be used to identify both conditions (22,23). Our observation that gait speed and grip strength were associated with low, medium, and high vertical impacts suggests that physical frailty is associated with reduced activity across all impacts, whereas our sarcopenia measure was only associated with high impacts. This suggests that other factors, related to muscle quality, may contribute to high impact activity. When patients with cardio-respiratory disease were excluded, gait speed was no longer related to high impacts, whereas a stronger association was observed with muscle force. A possible explanation being that patients with cardio-respiratory disease avoid high impact activity for reasons unrelated to sarcopenia, and their exclusion uncovers stronger underlying determinants of high impacts acting through sarcopenia.

Our finding that grip strength and gait speed were associated with reduced vertical impacts in all categories is consistent with previous studies. A longitudinal analysis identified grip strength to be associated with MVPA in men and women aged 60+ (10). In older men, gait speed and grip strength have been associated with MVPA (24). A recent longitudinal analysis identified gait speed to be predictive of change in amount of walking, independent of grip strength (25). In the present study, an independent influence of gait speed was observed, but not grip strength. Our finding that low ALMI was associated with reduced high impacts specifically, whilst muscle function was associated with reduced counts in all impact bands is consistent with the work of Rojer et al., who found an association between objectively-measured total daily PA and gait speed, but not muscle mass, in older adults (26).

High impacts have previously been associated with bone strength in our study population (18). Hence, increasing participation in higher impact activities, such as aerobics classes, may represent an effective means of improving bone strength and reducing fracture risk (27). Our findings suggest that in order to be successful, interventions need to address intrinsic factors which determine ability to generate high impacts, such as lower limb peak muscle force. In support of this conclusion, a previous meta-analysis found high impact PA alone was not effective at maintaining BMD at the hip or lumbar spine; other activity types, such as resistance training are required in combination with high impact activity to maintain BMD in postmenopausal women (16). Zhao et al. also found that a combination of resistance training and high impact or weight-bearing exercise increased BMD of the femoral neck and lower spine (28). The benefit of resistance training to improve muscle mass, strength, and physical performance is well established (4,29–31). As well as increasing bone strength, our findings may also reflect a positive impact of higher impact PA on sarcopenia and mobility. Hence, interventions designed to improve mobility and delay the onset of sarcopenia in older people may also benefit from inclusion of higher impact activities.

### Strengths and Limitations

The strengths of this study include the objective measurement of high impact PA by accelerometers. In addition, data were available for a wide range of variables allowing adjustment for a priori confounders. The availability of JM-derived variables as additional measures of lower limb muscle function is another strength.

The study populations were younger and more highly educated than the source population, which limits generalizability of our results to all community-dwelling older women. The population is female only; the relationship between sarcopenia and high impact PA has yet to be investigated in older men. Restricting JM analysis to those participants scoring ≥6 on SPPB meant we were unable to analyze the relationship between muscle function measures and high impact activity in participants with low physical function. This study is cross-sectional and therefore a causal relationship between low muscle function and high impact counts cannot be inferred; it is plausible that lack of high impact PA led to declines in muscle mass and function. We used the EWGSOP criteria to define sarcopenia as this is the most widely used criteria. However, a recent study of the performance of sarcopenia definitions found that the EWGSOP definition was worse at predicting physical and health-related outcomes (such as falls risk) compared to a simple strength test (32). The EWGSOP thresholds used are arbitrary. Finally, we used a threshold of 1.5 g to define high impact activity, due to the rarity of counts above this threshold (21); however, this is substantially lower than the 3.9 g threshold suggested to be beneficial to bone in premenopausal women (33).

## Conclusions

We investigated relationships between sarcopenia and habitual levels of PA categorized according to level of vertical impact, in a community-based population of older women. We found that gait speed and lower limb peak muscle force were independently associated with the number of high, potentially osteogenic, impacts during everyday activity. These findings underlie the importance of including exercises to increase gait speed and lower limb muscle force (such as resistance exercises) in PA interventions, intended to prevent osteoporosis through greater participation in high impact activities.

## Supplementary Material

Supplementary data is available at *The Journals of Gerontology, Series A: Biological Sciences and Medical Sciences* online.

Supplementary Information

## Funding

The VIBE study is funded by the Medical Research Council (grant ref MR/KO24973/1). A.H. is funded by the Wellcome Trust (grant ref 20378/Z/16/Z). C.L.G. is funded by a Clinician Scientist Fellowship from Arthritis Research UK (grant ref 20000). The COSHIBA cohort was originally funded through a Clinician Scientist Fellowship for EMC from Arthritis Research UK (grant ref 17823).

## Conflict of Interest

None Reported.

## Author Contributions

Study Design: K.H., E.M.C., J.H.T. Study Conduct: K.H., K.C.D., A.H., J.H.T. Data Collection: K.H., K.C.D., J.H.T. Data Analysis: A.H., C.L.G., J.H.T. Data Interpretation: A.H., C.L.G., J.H.T. Drafting manuscript: A.H., C.L.G., J.H.T. Revising manuscript and approving final version: A.H., C.L.G., K.H., K.C.D., E.M.C., J.H.T. A.H. takes responsibility for the integrity of data analysis.
