# Supplementary material for: Sarcopenia Is Negatively Related to High Gravitational Impacts Achieved From Day-to-day Physical Activity
Source: J Gerontol A Biol Sci Med Sci. 2017 Nov 22;73(5):652–9. doi: 10.1093/gerona/glx223 (PMC5905580; doi:10.1093/gerona/glx223)
Supplement: Supplementary Information [file glx223_suppl_supplementary_information.docx]

**Supplementary methods: Sarcopenia is is negatively related to high gravitational impacts achieved from day-to-day physical activity, Hartley et al**

Jumping Mechanography (JM)

Participant footwear was checked for suitability (flat soft soles; no heels) and removed if unsuitable. Prior to each assessment, the platform was calibrated (zero adjustment) and basic data input, *e.g.* age, gender, height. Vertebral osteoporosis was not considered a contraindication to JM. If participants expressed anxiety about their balance during the assessment, additional safety measures were considered, namely a) having an extra staff member present, and b) the staff member positioning themselves in front with arms out-stretched and forefingers pointing toward the participant to give reassurance (without adding any upward propulsion).

Clinic workers demonstrated and gave verbal instructions how to perform a 2-legged countermovement jump (2LJ) and one-legged hops (1LH). If necessary they stood beside the participant to assist with balance on landing. Participants could use their arms in any way that was comfortable and standard instructions were provided: *“I would like you to stand as still as you can please. When I instruct you to do so, I would like you to jump once as high as possible using both legs. Land on your forefoot and then stand as still as possible on both feet”*. For the single legged hopping test (1LH), participants were encouraged to start with their most comfortable or dominant leg and attempt 6-8 hops with the standard instructions: *“When I instruct you to begin, I would like you to hop on your forefoot, without landing on your heels using a stiff knee and follow the instructions as I provide them. Stand still to start on both legs”*. 2LJ and 1LH were each attempted three times, unless there was an error, in which case a maximum of four attempts was allowed. Participants remained standing between jumps/hops and recommenced when they were recovered and ready. Reasons were recorded if jumps/hops were not performed (unable/equipment failure/other). Clinic workers recorded adverse events. Peak power (kW) from the maximal valid 2LJ and the maximal peak force (kN) from all 1LH were extracted.

Dual X-ray Absorptiometry (DXA)

DXA scans were performed following a standard operating procedure and using a Lunar Prodigy scanner (GE Healthcare). Daily Quality Control (QC) checks were performed using the manufacturer’s phantom. All scans were checked and corrected, if necessary, for incorrect positioning of Regions of Interest (ROIs) using Lunar Prodigy enCORE software. Missing body mass on DXA images was coded during data cleaning. 20 participants returned within four weeks for repeat scans and within subject Coefficients of Variation (CVs) were 1.65% for FM and 1.48% for LM.

Peripheral Quantitative Computed Tomography (pQCT)

Consenting participants received a peripheral Quantitative Computed Tomography (pQCT) scan of the tibia at the 50% site using a Stratec XCT2000L scanner (Stratec Medizintechnik, Pforzheim, Germany). Scans were performed following a standard operating procedure. The right leg was scanned unless the participant had a recent fracture, noticeable tremor or had metal pins or plates in this leg, in which case the left leg was scanned if suitable. Each scan was checked and assigned an artefact grade ranging from 0 (no artefact) to 5 (large artefact- recognition of anatomical features not possible). Repeat scans were performed, if possible, for scans with a grade ≥3. All scans contributing to this analysis had a grade of 3 or less. Daily quality control checks were performed using the manufacturer’s phantom. 19 participants returned within four weeks for repeat pQCT; intra-subject CV for mCSA was 10.4%.

Covariates

Alcohol and smoking were self-reported in the study questionnaire. Participants were asked how many alcoholic drinks they had had in the past week and drinks were converted into units. Comorbidities were quantified by study questionnaire: participants were asked if they have or have ever had any disease potentially influencing PA participation (i.e. respiratory, cardiovascular, neurological and musculoskeletal diseases), and an additive score was produced from binary answers. Use of vitamin D/calcium supplements was also determined. Index of Multiple Deprivation (IMD) rank was taken from the English indices of deprivation 2015 (Department for Communities and Local Government, London,UK), using the 2013 postcodes. IMD rank, based on Lower Super Output Areas, ranked from 1 (most deprived) to 32,844 (least deprived), was categorized into quintiles with quintile one representing the most socioeconomically deprived participants.

Comparison with those in baseline cohort and not in the study population

Characteristics of those women in the 2015 study population, and those in the baseline COSHIBA study (2007-2009) but not in the 2015 study population were compared based on data collected in baseline questionnaires(1, 2) (except IMD which is described above); continuous variables using t-tests, categorical variables using chi-squared tests.

**Supplementary results**

Sensitivity analyses

Multivariable analyses were repeated using ≥2.0g to define high impacts. The median number of high impact counts were reduced to 12.0(5.4,27.4) (from 41.8(17.2,105.9) with threshold ≥1.5g). Results were essentially unchanged, with gait speed and peak force still the only exposures associated with high impacts in model 3, although peak force now had the stronger effect size compared to gait speed in the multivariate model [1.55(1.17,2.05) vs. 1.37(1.05,1.77)]. In further sensitivity analyses, we excluded women with neurological disease (n=18 for overall population and n=8 for the sub-population) or cardio-respiratory disease (n=92 and 55), participants with <3 days valid accelerometry data (n=30 and 20), those with missing body edges on DXA imaging (n=26 for the study population), and those with a pQCT error grade >2 (n=5 for the sub-population). Beta estimates were stable across these different analyses, with the exception of analyses excluding those with self-reported cardio-respiratory disease, after which there was less evidence for an association between gait speed and high impact counts in model 3 [1.32(0.97,1.78),p=0.07], whereas the relationship with peak force was strengthened [1.80(1.30,2.48)].

**Supplementary tables**

**Supplementary Table 1: Comparison of baseline characteristics (measured at enrolment in 2007-2009) of COSHIBA participants who were subsequently included in the main analyses (COSHIBA study sample) and the rest of the baseline population who were not included in these analyses (COSHIBA baseline population)**

|  | **COSHIBA original population**  **(not re-recruited)** | | **COSHIBA study sample**  **(re-recruited)** | |  |
| --- | --- | --- | --- | --- | --- |
|  | **N** | **Mean(SD)** | **N** | **Mean(SD)** | ***p*** |
| **Age** | 2820 | 73.1 (4.3) | 380 | 69.6 (2.9) | <0.01 |
| **Height (cm)** | 2507 | 160.0 (6.5) | 348 | 161.1 (6.5) | <0.01 |
| **Weight (kg)** | 2669 | 69.6 (13.5) | 372 | 68.3 (11.4) | 0.08 |
| **BMI (kg/m^2^)** | 2444 | 27.1 (5.0) | 341 | 26.3 (4.4) | <0.01 |
|  | **N** | **%** | **N** | **%** | ***p*** |
| **Smoker** |  |  |  |  | 0.15 |
| *Current* | 222 | 8.0 | 20 | 5.3 |  |
| *Past* | 1080 | 38.9 | 143 | 38.1 |  |
| *Never* | 1477 | 53.2 | 212 | 56.5 |  |
| **Weekly alcohol consumption** |  |  |  |  | <0.01 |
| *None* | 1254 | 45.1 | 120 | 31.7 |  |
| *Few glasses per week* | 1190 | 42.8 | 198 | 52.2 |  |
| *1 drink daily* | 240 | 8.6 | 44 | 11.6 |  |
| *>1 drink daily* | 86 | 3.1 | 16 | 4.2 |  |
| **Education/ qualifications** |  |  |  |  | <0.01 |
| *Up to age 16* | 2166 | 79.7 | 244 | 66.0 |  |
| *Up to age 18* | 254 | 9.3 | 70 | 18.9 |  |
| *Higher education* | 120 | 4.4 | 42 | 11.4 |  |
| *Other/ don’t know* | 179 | 6.6 | 14 | 3.8 |  |
| **IMD quintile^a^** |  |  |  |  | 0.01 |
| *1* | 587 | 20.9 | 56 | 14.7 |  |
| *2* | 552 | 19.7 | 82 | 21.6 |  |
| *3* | 573 | 20.4 | 92 | 24.2 |  |
| *4* | 561 | 20.0 | 64 | 16.8 |  |
| *5* | 535 | 19.1 | 86 | 22.6 |  |

N varies due to missing data for some variables at baseline.

COSHIBA: Cohort of Skeletal Health in Bristol and Avon, BMI: Body Mass Index, SD: Standard Deviation, *p: p* value for difference

^a^ IMD: Index of Multiple Deprivation. Quintiles based on IMD rank using postcode provided by NHS trace in 2013. 1= most deprived.

**Supplementary Table 2: Percentage difference in impact counts between sarcopenic and non-sarcopenic individuals for three clinical definitions of sarcopenia**

|  |  | **N=380** | **Model 1** | | **Model 2** | | **Model 3** | |
| --- | --- | --- | --- | --- | --- | --- | --- | --- |
|  |  | **N (%)** | **β (95% CI)** | ***p*** | **β (95% CI)** | ***p*** | **β (95% CI)** | ***p*** |
| **Low** | Grip strength <20kg | 148 (39.0) | 0.59 (0.48, 0.72) | <0.01 | 0.67 (0.55, 0.82) | <0.01 | 0.67 (0.54, 0.81) | <0.01 |
|  | Gait speed <0.8m/s | 67 (17.6) | 0.38 (0.29, 0.49) | <0.01 | 0.49 (0.38, 0.63) | <0.01 | 0.50 (0.39, 0.64) | <0.01 |
|  | ALMI ≤5.45kg/m^2^ | 45 (11.8) | 1.23 (0.90, 1.69) | 0.19 | 0.80 (0.59, 1.10) | 0.17 | 0.80 (0.59, 1.10) | 0.17 |
| **Medium** | Grip strength <20kg | 148 (39.0) | 0.51 (0.38, 0.67) | <0.01 | 0.56 (0.42, 0.75) | <0.01 | 0.54 (0.40, 0.72) | <0.01 |
|  | Gait speed <0.8m/s | 67 (17.6) | 0.35 (0.24, 0.49) | <0.01 | 0.43 (0.30, 0.61) | <0.01 | 0.43 (0.30, 0.62) | <0.01 |
|  | ALMI ≤5.45kg/m^2^ | 45 (11.8) | 1.01 (0.65, 1.55) | 0.98 | 0.66 (0.42, 1.03) | 0.07 | 0.68 (0.43, 1.06) | 0.09 |
| **High** | Grip strength <20kg | 148 (39.0) | 0.65 (0.49, 0.86) | <0.01 | 0.67 (0.50, 0.90) | <0.01 | 0.63 (0.47, 0.85) | <0.01 |
|  | Gait speed <0.8m/s | 67 (17.6) | 0.60 (0.42, 0.87) | <0.01 | 0.66 (0.45, 0.96) | 0.03 | 0.66 (0.45, 0.97) | 0.03 |
|  | ALMI ≤5.45kg/m^2^ | 45 (11.8) | 0.70 (0.46, 1.06) | 0.09 | 0.53 (0.34, 0.82) | <0.01 | 0.52 (0.34, 0.82) | <0.01 |

Exponentiated coefficients and confidence intervals are presented, representing the ratio of the geometric mean of impact counts between the groups (e.g. a beta of 1.23 represents a 23% increase in the geometric mean of low impact counts in the group with an ALMI below the sarcopenia threshold). Model 1: age-adjusted, Model 2: adjusted for age and height and weight for grip strength and gait speed or age and fat mass for ALMI, Model 3: adjusted as per model 2 plus Index of Multiple Deprivation, smoking, alcohol and comorbidities.

ALMI: Appendicular Lean Mass Index, CI: Confidence Interval, *p:p* value

Low impacts 0.5<0.5g<1.0g, medium impacts 1.0<g<1.5, high impacts≥1.5g

| **Model** | **r^2^** | **AIC** |
| --- | --- | --- |
| Base model^a^ | 0.09 | 832.25 |
| Base model + Gait speed | 0.16 | 816.94 |
| Base model + Chair rise time | 0.11 | 830.76 |
| Base model + Tandem balance | 0.10 | 833.66 |
| Base model + Grip strength | 0.11 | 829.87 |
| Base model + Peak force | 0.13 | 824.43 |
| Base model + Peak power | 0.10 | 832.00 |
| Base model + Muscle CSA | 0.10 | 833.04 |
| Base model + Gait speed + Peak force | 0.18 | 812.96 |
| Base model + All exposures | 0.18 | 821.19 |

**Supplementary table 3: Contribution of muscle function and mass variables to higher impact counts**

AIC: Akaike’s Information Criterion. CSA: Cross-Sectional Area

^a^Base Model: Age, height, weight, Index of Multiple Deprivation, comorbidities, smoking and alcohol.

**Supplementary References**

1.Clark EM, Gould V, Morrison L, et al. Randomized controlled trial of a primary care-based screening program to identify older women with prevalent osteoporotic vertebral fractures: Cohort for Skeletal Health in Bristol and Avon (COSHIBA).JBMR.2012;27(3):664-71.

2.Clark EM, Gould VC, Morrison L, et al. Determinants of fracture risk in a UK-population-based cohort of older women: a cross-sectional analysis of the Cohort for Skeletal Health in Bristol and Avon (COSHIBA).Age Ageing.2012;41(1):46-52.
